# Dual-Domain Reconstruction Network Incorporating Multi-Level Wavelet Transform and Recurrent Convolution for Sparse View Computed Tomography Imaging

**DOI:** 10.3390/tomography10010011

**Published:** 2024-01-16

**Authors:** Juncheng Lin, Jialin Li, Jiazhen Dou, Liyun Zhong, Jianglei Di, Yuwen Qin

**Affiliations:** 1 Institute of Advanced Photonics Technology, School of Information Engineering, Guangdong University of Technology, Guangzhou 510006, China; 2112103071@mail2.gdut.edu.cn (J.L.); jialinli127@163.com (J.L.); jiazhendou@gdut.edu.cn (J.D.); zhongly@gdut.edu.cn (L.Z.); qinyw@gdut.edu.cn (Y.Q.); 2 Guangdong Provincial Key Laboratory of Information Photonics Technology, Guangdong University of Technology, Guangzhou 510006, China; 3 Key Laboratory of Photonic Technology for Integrated Sensing and Communication, Ministry of Education, Guangdong University of Technology, Guangzhou 510006, China

**Keywords:** CT reconstruction, sparse view, dual-domain network, multi-level wavelet, convolutional long and short-term memory (Conv-LSTM), self-attention

## Abstract

Sparse view computed tomography (SVCT) aims to reduce the number of X-ray projection views required for reconstructing the cross-sectional image of an object. While SVCT significantly reduces X-ray radiation dose and speeds up scanning, insufficient projection data give rise to issues such as severe streak artifacts and blurring in reconstructed images, thereby impacting the diagnostic accuracy of CT detection. To address this challenge, a dual-domain reconstruction network incorporating multi-level wavelet transform and recurrent convolution is proposed in this paper. The dual-domain network is composed of a sinogram domain network (SDN) and an image domain network (IDN). Multi-level wavelet transform is employed in both IDN and SDN to decompose sinograms and CT images into distinct frequency components, which are then processed through separate network branches to recover detailed information within their respective frequency bands. To capture global textures, artifacts, and shallow features in sinograms and CT images, a recurrent convolution unit (RCU) based on convolutional long and short-term memory (Conv-LSTM) is designed, which can model their long-range dependencies through recurrent calculation. Additionally, a self-attention-based multi-level frequency feature normalization fusion (MFNF) block is proposed to assist in recovering high-frequency components by aggregating low-frequency components. Finally, an edge loss function based on the Laplacian of Gaussian (LoG) is designed as the regularization term for enhancing the recovery of high-frequency edge structures. The experimental results demonstrate the effectiveness of our approach in reducing artifacts and enhancing the reconstruction of intricate structural details across various sparse views and noise levels. Our method excels in both performance and robustness, as evidenced by its superior outcomes in numerous qualitative and quantitative assessments, surpassing contemporary state-of-the-art CNNs or Transformer-based reconstruction methods.

## 1. Introduction

Computed tomography (CT) is a non-invasive imaging technique based on X-ray projection, enabling the visualization of an object’s internal structure without destroying it. Therefore, it is widely applied in medical clinical diagnosis and non-destructive testing of composite materials in the industry [1,2]. However, X-rays pose a radiation hazard to the human body, and prolonged exposure to X-ray may lead to metabolic abnormalities and even induce cellular cancer. Hence, the as low as reasonably achievable (ALARA) principle [3] was proposed to advocate for the use of the lowest possible X-ray scanning dose without compromising CT imaging quality. One simple and effective method of reducing radiation dose is sparse view CT (SVCT) imaging, which decreases the number of X-ray projection views while also speeding up scanning [4]. However, SVCT reconstruction is a typical ill-posed problem, and the images reconstructed by conventional filtered back-projection (FBP) algorithm [5] suffer from severe artifacts and noise. To address the aforementioned issues in SVCT images, compressed sensing (CS) [6]-based iterative reconstruction algorithms have been proposed. For example, dictionary learning [7] and total variation [8] are introduced as the regularization terms in iterative algorithms to improve the quality of the SVCT image. Further, to mitigate the blurring issue caused by the total variation regularization term, ADS-POCS [9] and an adaptive-weighted TV minimization algorithm [10] have been proposed. The CS-based iterative algorithms achieved better reconstruction results compared to the FBP algorithm, but they are time-consuming due to multiple iterations of forward and backward projection in the calculation process. Moreover, the regularization terms and balance parameters need to be obtained from a large number of empirical experiments, and they are highly influenced by environmental factors, resulting in unstable reconstruction quality of the image.

In recent years, deep learning (DL) has achieved great success in the field of medical image processing [11,12,13]. Numerous DL-based SVCT reconstruction algorithms have emerged which have significantly improved the quality and speed of CT reconstruction. Early DL-based reconstruction algorithms mainly include image domain post-processing and sinogram domain pre-processing models. Image domain post-processing models can effectively remove streak artifacts in SVCT images using convolutional neural networks (CNNs), including, e.g., UNet-based FBPConvNet [14], densely connected blocks and deconvolution [15]-based DDNet [16], multi-level wavelet transform [17]-based MWNet [18], frequency-attention block and UNet-based model [19], and so on. On the other hand, sinogram domain pre-processing models aim to recover the projection data with missing views using CNNs, including UNet [20], generative adversarial network (GAN) [21], and others. However, the above two algorithms both lack consistency constraints on the data of another domain, resulting in secondary artifacts or subtle spurious structures in the reconstructed images.

Other DL-based reconstruction algorithms utilize multi-layer perceptron to implement mapping from a sensor domain to image domain data [22,23]. These models are designed not to require any expertise related to CT reconstruction. However, they suffer from an excessive number of parameters, rendering them impractical for reconstructing high-resolution CT images. In addition, the unfolding iterative models that integrate neural networks, such as LEARN [24] and ADMMBDR [25], are also classic DL-based reconstruction algorithms. In these models, CNNs are employed to update the regularization term of the iterative model or implement subproblem mapping solutions during the iterative process, aiming to reduce computational complexity and enhance reconstruction quality. Nevertheless, these models also entail significant computational overhead, making them still time-consuming compared to the above DL-based reconstruction algorithms.

Furthermore, to reduce the computational overhead and facilitate the interaction of feature information between sinograms and CT images, hybrid domain reconstruction networks have attracted extensive research interests in recent years. They compensate for the drawbacks of single-domain data processing models while achieving constraints on the consistency of sinograms and CT images. For example, CNNs and recurrent data fidelity layer-based DuDoDR-Net [26], 3D UNet-based HDNet [27], densely connected block, and multilevel wavelet transform-based DuMWNet [28]. Although CNNs-based hybrid domain networks have demonstrated excellent reconstruction performance, the local convolution operation of CNNs limits the size of the perceptual field, resulting in its inability to better model the contextual long-range dependencies between feature maps, thus limiting its feature extraction ability.

Vision Transformer (VIT) is a new feature extraction module in computer vision which can capture global feature information through a self-attention mechanism in Transformer and exhibits a more powerful feature extraction ability compared to CNNs [29]. Consequently, various Transformer-based SVCT hybrid domain reconstruction networks have emerged. For example, Wang et al. [30] proposed Swin-Transformer (SwinT)-based DuDoTrans, enhancing the model’s recovery ability by capturing global feature information from sinograms and CT images. Li et al. [31] proposed a DDPTransformer, which enhanced the ability of the dual-domain network to capture edge feature information by integrating the parallel SwinT with different patch segmentation schemes. Pan et al. [32] proposed a MIST, which further enhanced the quality of CT images through SwinT after the iterative optimization of sinograms and CT images using multiple UNets. In addition, for the iterative reconstruction model, Xia et al. [33] also proposed a regularization term update method based on Transformer and CNNs to enhance the ability of the iterative model to extract global prior information of CT images. To reduce the computational parameters and overhead, these Transformer-based reconstruction networks employ a simple codec structure, which directly extracts features through the SwinT block after a convolution operation. They both ignore the extraction and recovery of feature information at different depth levels. The simple single-branch codec structure also introduces a network bias towards recovering the low-frequency components of the image, ignoring the recovery of a detailed image structure to some extent.

To address the above problem, inspired by the dual-domain network structure and wavelet domain-based learning methods [18,34], a dual-domain reconstruction network incorporating multi-level wavelet transform and recurrent convolution is proposed for SVCT imaging. Interpolated sinograms and CT images are simultaneously optimized by two wavelet transform-based sub-networks to achieve end-to-end CT reconstruction. Our method is able to enhance the recovery of distinct frequency information in sinograms and CT images by learning in the frequency domain instead of the original image domain, and our contributions are summarized as follows:

(1) A new CT reconstruction model is proposed, wherein multi-level wavelet transform and recurrent convolution unit construction are integrated. To accurately correct the errors in each frequency component of the interpolated sinograms and remove the artifacts in different directions in each frequency component of the CT images, multi-level wavelet transform is employed to decompose the sinograms and CT images into distinct frequency components, which are subsequently individually recovered through separate network branches.
(2) To capture the global redundant texture information of sinograms and the global artifact features in CT images in distinct frequency components, a recurrent convolution unit (RCU) embedded with convolutional long and short-term memory (Conv-LSTM) is proposed. The RCU consists of a basic feature extraction module “3 × 3 convolution-batch normalization-ReLU activation function” (CBR) and a Conv-LSTM, where CBR is used to integrate the output features of the previous layer and adjusts the number of channels, while the Conv-LSTM weights the hidden and memory state features from the output of the previous RCU layer into the output of the current layer by combining forget gate, input gate, and output gate operations to model long-distance dependencies between the feature map in different layers, as well as the information flow of contextual textures in distinct frequency dimensions.
(3) In the high-frequency component recovery network branch, an improved multi-level frequency feature normalization fusion (MFNF) block is designed to assist in the recovery of high-frequency components by aggregating low-frequency components through the self-attention-based normalization strategy. Further, the recovery of high-frequency feature information is enhanced by combining an adaptive channel soft thresholding function (ACSTF) to filter out noise and useless features in the channel dimension.
(4) In the image domain loss function, an additional edge loss regularization term based on the Laplacian of Gaussian (LoG) is designed to improve the fidelity and authenticity of high-frequency edge details and mitigate the structural blurring caused by mean squared error (MSE) loss function.

## 2. Materials and Methods

### 2.1. Principles of CT Reconstruction

In CT detection equipment, the intensity of the X-ray source acquired by the detectors follows the Beer–Lambert Law of attenuation [35]. It states that X-ray energy is absorbed by the object, resulting in an attenuation of its own intensity after penetrating the object. For a two-dimensional object cross-section (*x*, *y*), different locations have different attenuation coefficients *f*(*x*, *y*). When an X-ray of incident intensity *I*_0_ penetrates the object, the final detected photon intensity I can be expressed as

(1) $$I=I_{0}e^{-\int_{L}f\left(x,y\right)dl}$$

where *L* is the path of the X-rays through the object and $\int_{L}f(x,y)dl$ is the linear decay superimposed integral of the X-rays along the path of *L*. The resulting projection data *g* can therefore be expressed as

(2) $$g=-\ln\left(\frac{I}{I_{0}}\right)=\int_{L}f\left(x,y\right)dl$$

And CT reconstruction can be expressed as the following linear inverse problem

(3) g=Af+n

where *A* is the system projection matrix, and *n* is noise. In SVCT reconstruction, the reconstruction process becomes an ill-posed problem due to the sampling views not satisfying Nyquist’s theorem and the presence of systematic noise, and the reconstructed CT images will suffer from streak artifacts.

### 2.2. Network Structure

**Figure 1:**
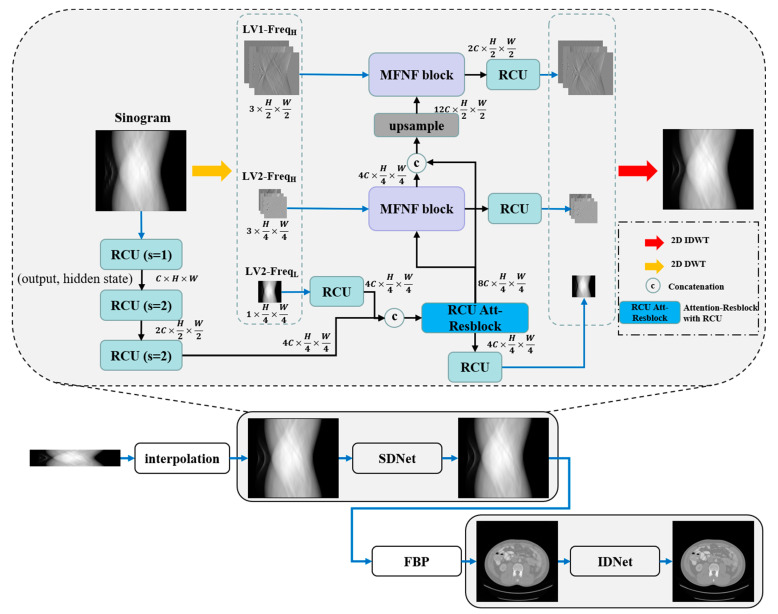
Architecture of the proposed dual-domain reconstruction network incorporating multi-level wavelet transform and recurrent convolution.

The overall architecture of the dual-domain reconstruction network incorporating multi-level wavelet transform and recurrent convolution is illustrated in Figure 1. The architecture comprises a sinogram domain network (SDNet), an image domain network (IDNet), and an intermediate FBP embedding layer, where the two single-domain sub-networks have the same structure. Sparse view sinograms are first interpolated using bilinear interpolation, and then the sinograms and reconstructed CT images are further optimized through two wavelet transform-based single-domain networks. In the two single-domain networks, the input images are decomposed into sub-images of distinct frequency bands using discrete wavelet transform (DWT), including low-frequency image components and high-frequency image components of different directions. Subsequently, these components are fed into separate network branches for recovery. In the low-frequency component recovery network branch, the original input images are first downsampled using RCU, which are then concatenated with the low-frequency components, and further the fusion features are optimized by the RCU-Att-Resblock that combines the self-attention mechanism with the RCU. Finally, the clean low-frequency sub-image is obtained by the RCU. In the different high-frequency component recovery network branches, dense features from the low-frequency band components are progressively fused by the MFNF block to enhance the recovery of high-frequency components. Finally, 2D inverse discrete wavelet transform (IDWT) is used to reconstruct the recovered components of each frequency band as sinograms or CT images. The initial number of expansion channels (C) for both SDNet and IDNet is set to 32, and the numeric symbols (2C, 4C, 8C, 12C) are expanded multiples of the number of channels in the different layers.

#### 2.2.1. Discrete Wavelet Transform (DWT)

The projection data in sinograms as well as the artifacts in CT images are characterized by significant directionality and global distribution. Therefore, we use DWT with directional filters to effectively decompose the different directional components of the noises and artifacts in the images and then learn the distribution regularities of these features through networks to enhance the recovery of SVCT images. In this work, 2D discrete Haar wavelet transform is used to decompose the images. It consists of four different directional filters (*f_LL_*, *f_LH_*, *f_HL_*, *f_HH_*), which are defined as [17]

(4) $$f_{LL}=\left[\begin{array}{cc} 1 & 1\\ 1 & 1 \end{array}\right],\;f_{LH}=\left[\begin{array}{cc} -1 & -1\\ 1 & 1 \end{array}\right],\;f_{HL}=\left[\begin{array}{cc} -1 & 1\\ -1 & 1 \end{array}\right],\;f_{HH}=\left[\begin{array}{cc} 1 & -1\\ -1 & 1 \end{array}\right]$$

For the discrete Haar wavelet decomposition of an image *x*, we need to utilize the above four filters to convolve with the original image and then downsample the results. Finally, we can obtain four wavelet sub-band images. These decomposed images include the low-frequency component *x_LL_*_1_((*f_LL_*⊗*x*) ↓2), the horizontal high-frequency component *x_LH_*_1_((*f_LH_*⊗*x*) ↓2), the vertical high-frequency component *x_HL_*_1_((*f_HL_*⊗*x*) ↓2), and the diagonal high-frequency component *x_HH_*_1_((*f_HH_*⊗*x*) ↓2). The further two-level wavelet transform continues by applying four filters to convolve with the low-frequency component *x_LL_*_1_ and then downsample the result to obtain the corresponding wavelet sub-band components. In this work, a two-level wavelet transform is used for the decomposition of the images, and the differentiable DWT and IDWT modules in PyTorch are implemented through the “pytorch_wavelets” library [36]. As illustrated in Figure 1, the inputs to the three branches of the network are the two-level low-frequency component image *x_LL_*_2_ (LV2-freq_L_), two-level high-frequency component images {*x_LH_*_2_, *x_HL_*_2_, *x_HH_*_2_} (LV2-freq_H_), and one-level high-frequency component images {*x_LH_*_1_, *x_HL_*_1_, *x_HH_*_1_} (LV1-freq_H_), respectively.

#### 2.2.2. Recurrent Convolution Unit (RCU)

**Figure 2:**
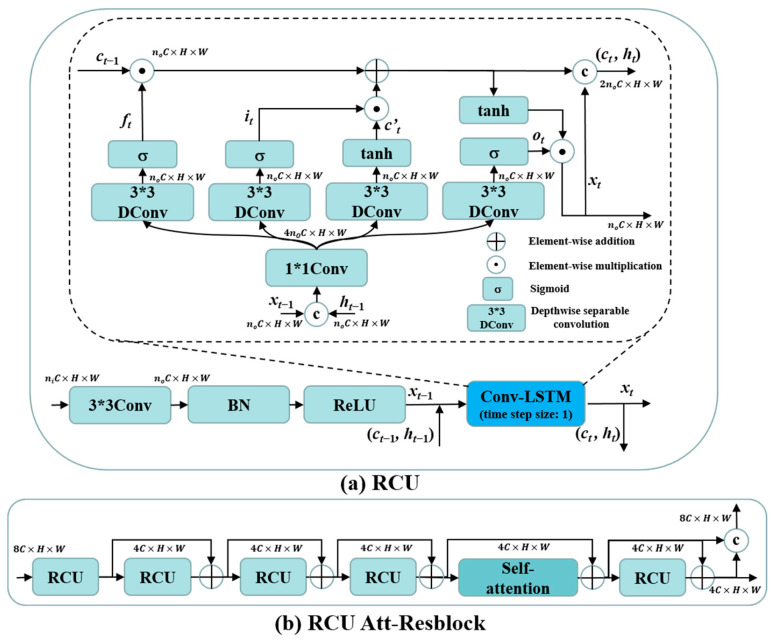
(**a**) Architecture of the RCU; (**b**) architecture of the RCU Att-Resblock.

Since the projection data are acquired by scanning around the target with X-ray, the sinogram information in different positions exhibits complementary characteristics, which makes the full view sinograms redundant with texture information. Therefore, to characterize the global distribution of the decomposed sinograms and the global artifacts in decomposed SVCT images, a recurrent convolution unit (RCU) was designed to enhance the feature extraction ability of the network by embedding the Conv-LSTM [37] after the CBR. As illustrated in Figure 2a, we utilize the Conv-LSTM to recursively calculate the previous moment memory and hidden states, capturing global feature information and modeling the information flow of contextual textures in distinct frequency dimensions. The *n_i_* and *n_o_* in Figure 2a are multiples of the number of input and output channels, respectively, and the number of output channels for RCU are adjusted by a 3 × 3 convolution (Conv). The specific values of *n_i_* and *n_o_* for the RCU in different layers depend on multiples of the number of input and output channels (C) for RCU shown in Figure 1 and Figure 2b. To reduce the number of parameters in the Conv-LSTM, we replace the original 3 × 3Conv by combining 1 × 1Conv and 3 × 3 Depthwise separable convolution (DConv). Assuming that the output feature after CBR operation is *x_t_*_−1_ and the hidden and memory states output by the RCU in the previous layer are *h_t−_*_1_ and *c_t_*_−1_, we first concatenate *h_t_*_−1_ with *x_t_*_−1_ in the channel dimension to obtain (*h_t_*_−1_, *x_t_*_−1_). Subsequently, the forget gate *f_t_*, input gate *i_t_*, and output gate *o_t_* operation processes in Conv-LSTM can be expressed as

(5) $$\begin{array}{l} f_{t}=\sigma [W_{f}⊗(x_{t-1},h_{t-1})+b_{f}],\\ i_{t}=\sigma [W_{i}⊗(x_{t-1},h_{t-1})+b_{i}],\\ o_{t}=\sigma [W_{o}⊗(x_{t-1},h_{t-1})+b_{o}] \end{array}$$

where *σ* and ⊗ are the sigmoid activation function and the “1 × 1Conv-3 × 3DConv” operation process, respectively. *W* and *b* are the different convolution kernels and bias parameters, respectively. The final output of the Conv-LSTM can be expressed as

(6) $$\begin{array}{l} c_{t}^{\prime }=\tanh[W_{c}⊗(x_{t-1},h_{t-1})+b_{c}],\\ c_{t}=f_{t}⊙c_{t-1}+i_{t}⊙c_{t}^{\prime },\\ h_{t}=o_{t}⊙\tanh(c_{t}) \end{array}$$

where ⊙ is the element-wise multiplication operation, $c_{t}^{\prime }$ is the candidate memory states of the input features at the current moment, and *h_t_* is the output features (*x_t_*) and hidden states of the RCU at the current moment. The final weighted memory states, *c_t_*, from both the previous and current moments exert a proportional influence on the current moment’s output through the control of *o_t_*. As the output of *o_t_* approaches 1, more information about past features is preserved in the output of RCU. This mechanism allows for the modeling of long-distance dependencies between feature maps in different layers through the RCU. Specifically, given that the initial input sinogram or CT image of the network is a single-frame image rather than continuous time-series images, we use only one Conv-LSTM cell in the RCU, corresponding to a time step size of 1. Meanwhile, this operation prevents the excessive increase in the number of model parameters and running time resulting from stacking too many Conv-LSTM cells in a single RCU. Further, the structure of RCU-Att-Resblock is shown in Figure 2b, including four RCU blocks and a self-attention block [38], and their outputs are connected by residual learning to enhance the extraction capability of this branch for low-frequency features.

#### 2.2.3. Multi-Level Frequency Feature Normalization Fusion (MFNF) Block

**Figure 3:**
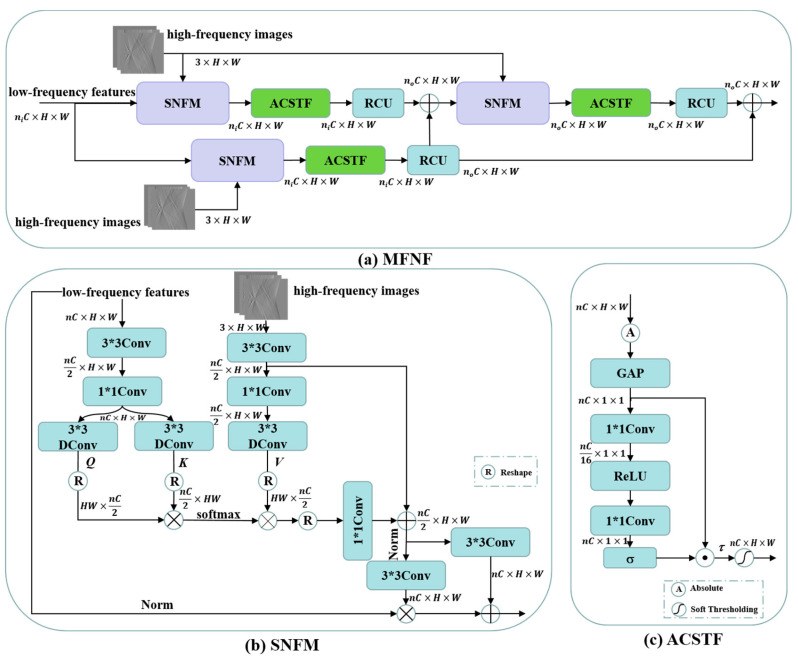
(**a**) Architecture of the MFNF block; (**b**) architecture of the SNFM; (**c**) architecture of the ACSTF.

High-frequency components are characterized by greater sparsity in spatial dimensions. If high-frequency components and low-frequency components are fused by simple summation or concatenation only, it will largely suppress the high-frequency information components and thus affect the recovery of high-frequency components. Different to the high- and low-frequency feature concatenation fusion approach of the MWNet [18] and DuMWNet [28], we construct a self-attention-based frequency feature normalization fusion module (SNFM) in the multi-level frequency feature normalization fusion (MFNF) block by combining DConv-based self-attention with a frequency region normalization strategy [34]. It can effectively aggregate the feature information of low-frequency components into high-frequency components to enhance the recovery of high-frequency components. To further filter out the useless frequency feature components or noise components, we also construct an adaptive channel soft thresholding function (ACSTF) based on the work of deep residual shrinkage networks [39] instead of simply using the ReLU activation function. The overall structure of the MFNF block is shown in Figure 3a, which enhances the delivery of high-frequency feature information flowing through residual learning. The *n_i_* and *n_o_* in Figure 3a are the multiples of the number of input and output channels, respectively. The specific value of *n_i_* depends on the multiples of the number of input channels (C) for the low-frequency features in the MFNF block shown in Figure 1, and *n_o_* depends on the multiples of the number of output channels (C) in the MFNF block. The *n* in Figure 3b,c is the multiple of the number of channels for the low-frequency feature (*n_i_* in Figure 3a) or the residual fusion features of low-frequency features and high-frequency components (*n_o_* in Figure 3a).

The structure of SNFM is illustrated in Figure 3b. Similar to the process of self-attention calculation, this module first aggregates the contextual information of low-frequency components by 3 × 3Conv and 1 × 1Conv, then extracts Query (*Q*) and Key (*K*) in the low-frequency feature branches by 3 × 3Donv, and finally calculates the features after matching the dot product of *Q* and *K* by softmax function to obtain the attention score maps *W*. It is further multiplied with the Value (*V*) that was obtained by encoding the high-frequency components through a 3 × 3DConv to obtain the attention feature *A*

(7) $$\begin{array}{l} W=\mathrm{softmax}(\frac{QK^{T}}{\sqrt{d_{k}}}),\\ A=WV \end{array}$$

the attention score maps describe the correlation between the global texture information of low-frequency features, so we recover the high-frequency feature components by aggregating the low-frequency features from the output of the underlying network branch. The advantage of using a DConv-based self-attention calculation is that the global contextual attention maps can be implicitly encoded by calculating cross-covariances across channels, making it suitable for directly processing high-resolution feature maps. It thus alleviates the problems of high spatial self-attention computation overhead and memory complexity in VIT [40] and SwinT [41], and self-attention learning from 2D space can reduce the features loss in downscaling feature maps from 2D to 1D. Further, to address the problem of large differences in high- and low-frequency feature distributions, the initial aggregated high- and low-frequency features are normalized separately by parameter-free position normalization [42], which preserves the structural information while homogenizing the feature distribution, thereby reducing the magnitude of feature fusion. Therefore, the 3 × 3Conv operation is performed on the residual attention feature *A* to generate normalized modulation parameters. The parameters are then used to modulate the complete structural information in the low-frequency feature components to generate high-frequency information for recovering the CT images, ultimately achieving an effective alignment fusion of the high- and low-frequency components

(8) $$x_{H}=\gamma_{H}\frac{x_{L}-\mu_{L}}{\sigma_{L}}+\beta_{H}$$

where *x_H_* is the output of the SNFM, *x_L_* is the normalized low-frequency feature of the input, and *µ_L_*, *σ_L_* are the mean and standard deviation of *x_L_* along the channel dimension. *γ_H_*, *β_H_* are the normalized modulation parameters corresponding to the output of the two 3 × 3Conv.

Further, the ACSTF is illustrated in Figure 3c, where it adaptively learns a set of thresholds through the Squeeze-and-Excitation (SE) channel attention mechanism and then combines a soft thresholding function to set the useless features to zero while shrinking other features towards zero to achieve signal denoising and filtering redundant features. The process of calculating the soft thresholds *τ* can be expressed as

(9) $$\begin{array}{l} z=\mathrm{FC}\left\{\mathrm{ReLU}\left[\mathrm{FC}\left(\mathrm{GAP}\left(\left|x_{H}\right|\right)\right)\right]\right\},\\ \tau =\mathrm{Sigmoid}\left(z\right)⊙\mathrm{GAP}\left(\left|x_{H}\right|\right) \end{array}$$

where GAP is the maximum average pooling layer, and the absolute value operation | | ensures that the soft thresholds are non-negative. In FC, we replace the fully connected layer in the original SE by 1 × 1Conv. The soft thresholding function is further combined to obtain the output features *y* of ASTF

(10) $$y=\{\begin{array}{c} \begin{array}{cc} x_{H}-\tau , & x_{H}>\tau \end{array}\\ \begin{array}{cc} 0, & -\tau \leq x_{H}\leq \tau \end{array}\\ \begin{array}{cc} x_{H}+\tau , & x_{H}<-\tau \end{array} \end{array}$$

#### 2.2.4. Loss Function

In the SDNet, the mean absolute error (MAE) is used as the constrained loss function

(11) $$L_{\mathrm{sino}}={\left\|S-S_{ref}\right\|}_{1}$$

where *S* denotes the sinograms recovered by SDNet, and *S_ref_* denotes the full view reference sinograms, respectively. In the IDNet, the recovery of CT images is constrained using the MSE loss function

(12) $$L_{img}={\left\|I-I_{ref}\right\|}_{2}^{2}$$

where *I* and *I_ref_* denote the CT images reconstructed by the IDNet and the reference CT images, respectively. Further, to alleviate the problem that the MSE loss function tends to cause over-smoothing of the reconstructed structure, based on the Laplacian of Gaussian (LoG) [43] and MAE loss, an additional edge loss is designed as a regularization term to improve the fidelity and authenticity of high-frequency component details, which is defined as

(13) $$L_{edge}={\left\|\nabla^{2}G_{\sigma }⊗I-\nabla^{2}G_{\sigma }⊗I_{ref}\right\|}_{1}$$

where *I* and *I_ref_* denote the reconstructed image and the reference image, respectively. ⊗ is the convolution operation, and $\nabla^{2}G_{\sigma }$ represents the second order derivative of the Gaussian kernel. The second order derivative of a 2D Gaussian distribution function with mean 0 and standard deviation *σ* can be expressed as the following equation

(14) $$\nabla^{2}G_{\sigma }(x,y)=-\frac{1}{\pi \sigma^{4}}\left(1-\frac{x^{2}+y^{2}}{2\sigma^{2}}\right)e^{-\frac{x^{2}+y^{2}}{2\sigma^{2}}}$$

where a LoG with a 5 × 5 convolutional kernel is employed to extract the edge features of the images. Regarding the edge loss function, the output images of the network and the reference images are convolved by a convolutional kernel with a fixed parameter that approximates the discrete LoG distribution. The backpropagation of the gradient is subsequently implemented using MAE loss and the Adam optimizer [44] in PyTorch. The total loss function of the dual-domain network is defined as

(15) $$L=L_{\mathrm{sino}}+L_{\mathrm{img}}+\alpha L_{\mathrm{edge}}$$

where the weight parameter α for the edge loss function is set to 0.02.

## 3. Results and Analysis

### 3.1. Dataset and Simulation Setup

The “Low Dose CT Image and Projection Data (LDCT-and-Projection-data)” dataset [45] provided by the Mayo Clinic was utilized for generating simulated sparse view sinograms and CT images, serving both as training and evaluation data for our proposed CT reconstruction network. The full dose Mayo data were scanned under the protocol of 120 kVp and 225 effective mAs (500 mA/0.45 s). In total, 24 patients (8 chest cases, 8 abdominal cases, and 8 head cases) were randomly selected for the training set, while the validation set comprised 3 patients (1 chest case, 1 abdominal case, and 1 head case), and the test set included 3 patients (1 chest case, 1 abdominal case, and 1 head case). Finally, the total number of training sets is 4025, the total number of validation sets is 413, and the total number of test sets is 456. The resolution of the CT image is 512 × 512.

Simulated sparse view sinograms were generated using fan beam X-ray geometry, distributing a total of 512 scan views uniformly across 360°. The distance from the X-ray source to the detector array is 1280 mm, and the distance from the X-ray source to the rotation center is 640 mm. The detector array consists of 720 detector units, with each unit measuring 1 mm in length. Projection views are configured at 128, 64, and 32 to simulate CT reconstruction scenarios involving varying numbers of sparse views. The differentiable FBP algorithm in PyTorch is implemented using the “ctlib” library [46]. Additionally, the model’s resilience to photon noise is assessed by introducing combined “Gaussian + Poisson” noise to the simulated sparse view sinograms [10,31]

(16) s=exp(−s/MAX(s))

(17) $$s=s+I_{0}*P(s)+I_{0}*G(m,var/I_{0})$$

(18) $$s=-\log(s/I_{0})*\mathrm{MAX}(s)$$

where *s* is the sparse view sinogram, MAX(*s*) is the max value of the sparse view sinogram, *G*(*m*, *var/I*_0_) is the Gaussian noise with mean *m* = 0 and variance *var* = 0.05, and *P*(*s*) is the Poisson noise with average photon count *I*_0_ = 5 × 10^6^ This noise simulation aims to replicate the noise inherent in the sensor-generated data during the process of acquiring projection data.

The PyTorch framework was employed for the implementation of our model. The Adam optimizer was used for model training, with the momentum terms *β*_1_ and *β*_2_ set to 0.9 and 0.999, respectively. The initial learning rate for the model training was set to 0.001, which was gradually reduced to 0.0005 using a multi-step decay strategy. The training process encompassed 25 epochs, and a mini-batch size of 1 was chosen. All experiments were conducted on a server with a 24G NVIDIA RTX 3090 GPU, an Intel(R) Xeon(R) Gold 5218 CPU @ 2.10 GHz, and 256 GB of RAM.

### 3.2. Qualitative Evaluation

In this work, to verify that our model has stronger reconstruction and generalization performance, previous analytic reconstruction algorithm, and CNNs or Transformer-based SVCT reconstruction networks are used for comparison with our method, including FBP [5], MWNet [18], DuDoTrans [30], DDPTransformer [31], MIST [32], and RegFormer [33]. Specifically, this section compares the 2D reconstructed slices, the enlarged region of interest (ROI) of the corresponding slices, the absolute difference images relative to the reference image, and the corresponding relative root mean square error (rRMSE) results of the different methods with 128, 64, and 32 projection views.

**Figure 4:**
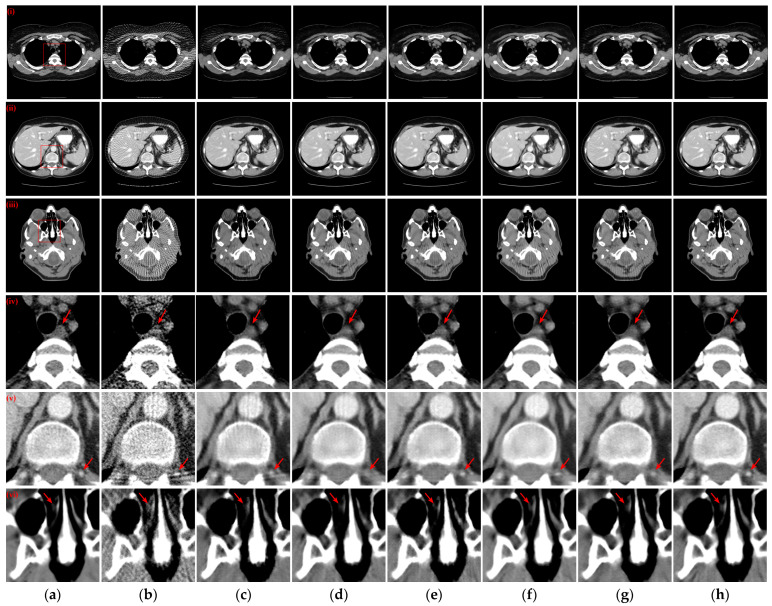
The SVCT reconstruction results from different methods with 128 projection views. (**a**) The reference image; (**b**) FBP; (**c**) MWNet; (**d**) DuDoTrans; (**e**) DDPTransformer; (**f**) MIST; (**g**) RegFormer; (**h**) Ours. (**i**)–(**iii**) are the chest CT, abdominal CT, and head CT, respectively, and (**iv**)–(**vi**) correspond to the enlarged ROIs marked by the red boxes in the reference images above. The display window is [−160, 240] HU.

Three reconstructed CT slices of different human tissues were randomly selected from the test set to qualitatively compare the reconstruction performance of all models. Figure 4 presents the reconstruction outcomes obtained using different methods with 128 projection views and the ROIs in the reference images, indicated by red boxes, are depicted, and the enlarged ROIs corresponding to the reconstruction results from various methods are displayed below the reconstructed CT images. From the observations in the reconstructed CT images of Figure 4(i)–(iii), it becomes evident that the results obtained through the FBP algorithm exhibit streak artifacts and a decline in contour structure quality. While the post-processing method generally manages to eliminate most of the streak artifacts, the visual fidelity of the images often falls short compared to the hybrid domain and iterative reconstruction network. The latter two approaches, which enforce recovery constraints on both sinograms and CT images, yield reconstruction results that closely resemble the reference image. Furthermore, our method demonstrates even greater artifact reduction than other hybrid domains and unfolding iterative method. Examining the enlarged ROIs comparison results in Figure 4(iv)–(vi) reveals that MIST and RegFormer reconstruct more comprehensive structures than other DL-based comparison algorithms, yet they still experience structural blurring in certain regions. In contrast, our method significantly enhances the sharpness of edge contours, resulting in improved alignment with the reference images, particularly for soft tissue and bone structures. In particular, due to the small amount of data for the head CT images in the original dataset, different methods underfit the head CT reconstruction results in Figure 4(iii,vi), resulting in more artifact information. Nevertheless, our method was still able to remove more artifacts and preserve more details.

**Figure 5:**
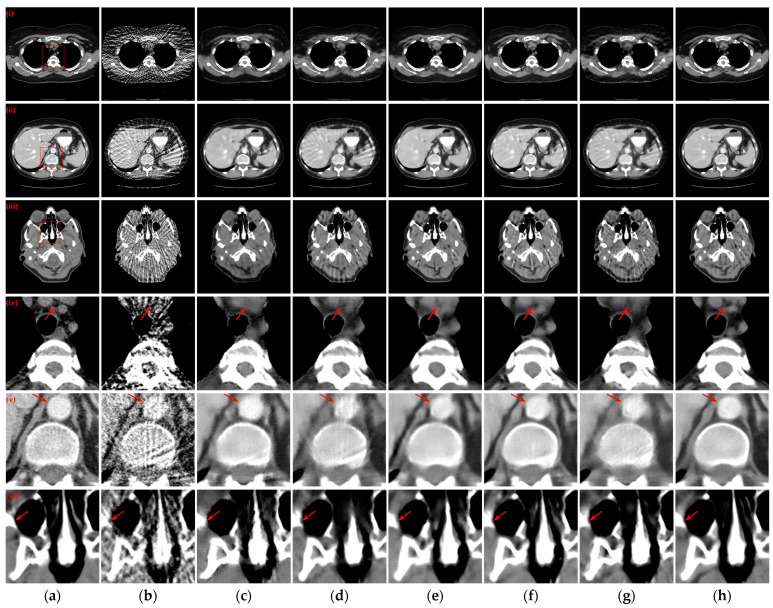
The SVCT reconstruction results from different methods with 64 projection views. (**a**) The reference image; (**b**) FBP; (**c**) MWNet; (**d**) DuDoTrans; (**e**) DDPTransformer; (**f**) MIST; (**g**) RegFormer; (**h**) Ours. (**i**)–(**iii**) are the chest CT, abdominal CT, and head CT, respectively, and (**iv**)–(**vi**) correspond to the enlarged ROIs marked by the red boxes in the reference images above. The display window is [−160, 240] HU.

Figure 5 illustrates the reconstruction outcomes obtained using different methods with 64 projection views. Similarly, the enlarged ROIs corresponding to the reconstruction results of the different methods are displayed below the reconstructed CT images. The observations from Figure 5(i)–(iii) indicate that as the number of projection views decreases, the efficacy of artifact suppression diminishes for the post-processing method, and blurring becomes more prominent in the results of the hybrid domain or iterative reconstruction network. Further insights from the enlarged ROIs in Figure 5(iv)–(vi) highlight that our method continues to yield superior qualitative outcomes, successfully eliminating artifacts while achieving a more comprehensive structure and well-defined edge contours.

**Figure 6:**
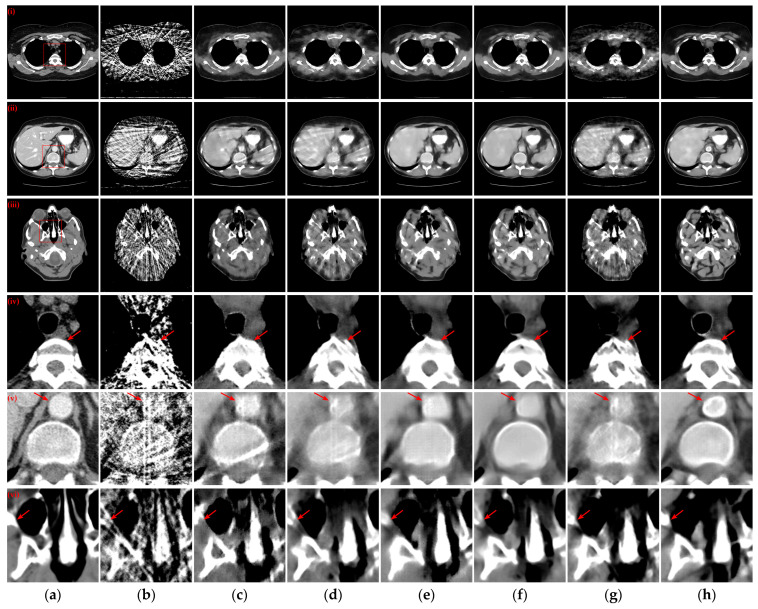
The SVCT reconstruction results from different methods with 32 projection views. (**a**) The reference image; (**b**) FBP; (c) MWNet; (**d**) DuDoTrans; (**e**) DDPTransformer; (**f**) MIST; (**g**) RegFormer; (**h**) Ours. (**i**)–(**iii**) are the chest CT, abdominal CT, and head CT, respectively, and (**iv**)–(**vi**) correspond to the enlarged ROIs marked by the red boxes in the reference images above. The display window is [−160, 240] HU.

To examine the scenario with a further reduction in projection views to 32, the reconstructed CT images obtained by various methods and corresponding enlarged ROIs are presented in Figure 6. The FBP algorithm struggles to reconstruct the complete and clear structure with only 32 projection views. Among the compared DL-based reconstruction networks, all methods except MWNet, DuDoTrans, and RegFormer exhibit better artifact suppression and reduced structural blurring. Our method, however, goes a step further in addressing the structural blurring issue, resulting in reconstruction outcomes that closely align with the reference images. Further analysis of the enlarged ROIs in Figure 6(iv,v) reveals a noteworthy pattern: almost exclusively, our method manages to reconstruct the complete structure and edge contours more closely to the reference images. In contrast, all other DL-based reconstruction methods lose more structural information.

**Figure 7:**
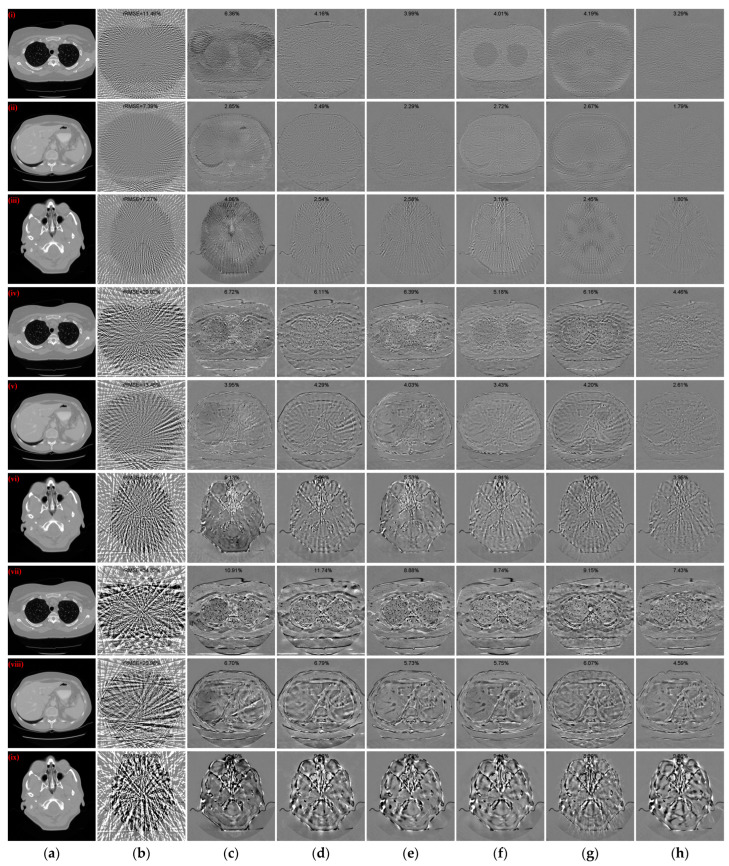
Absolute difference images relative to the reference images with different projection views. (**a**) The reference image; (**b**) FBP; (**c**) MWNet; (**d**) DuDoTrans; (**e**) DDPTransformer; (**f**) MIST; (**g**) RegFormer; (**h**) Ours. (**i**)–(**iii**) correspond to the results with 128 projection views, (**iv**)–(**vi**) correspond to the results with 64 projection views, and (**vii**)–(**ix**) correspond to the results with 32 projection views. The display window for reference images is [−1000, 800] HU, and the display window for absolute difference images is [−180, 180] HU.

To further evaluate the qualitative results of the reconstructed CT images, the absolute difference images of the reconstructed images relative to the reference images for different methods with different sparse projection views were used for comparative analyses. Simultaneously, the rRMSE between the reconstructed image and the reference image is calculated, which is defined as the following equation

(19) $$\mathrm{rRMSE}=\frac{{\left\|I-I_{ref}\right\|}_{2}}{{\left\|I_{ref}\right\|}_{2}}\times 100\%$$

where *I* and *I_ref_* denote the reconstructed image and the reference image, respectively. The rRMSE value is smaller to indicate a smaller error between the reconstructed CT image and the reference image. Figure 7 illustrates the absolute difference images of the reconstructed images obtained using different methods relative to the reference CT images in Figure 4(i)–(iii), Figure 5(i)–(iii), and Figure 6(i)–(iii), respectively. With less views, the absolute difference images obtained from each method are worse. But our method generates the smallest difference compared to other methods, so that the reconstructed result is closer to the original image. In terms of both 128 views and 64 views, our method achieved the lowest rRMSE value on different human tissues.

### 3.3. Quantitative Evaluation

To demonstrate the effectiveness of our method directly, three quantitative metrics are used to evaluate the quality of the reconstructed CT images: structural similarity (SSIM) [47], root mean square error (RMSE), and peak signal-to-noise ratio (PSNR). SSIM calculates the degree of similarity between the reconstructed image and the reference image by combining three factors: brightness, contrast, and structure

(20) $$\mathrm{SSIM}=\frac{\left(2\mu_{I}\mu_{I_{ref}}+c_{1}\right)\left(2\sigma_{I,I_{ref}}+c_{2}\right)}{\left(\mu_{I}^{2}+\mu_{I_{ref}}^{2}+c_{1}\right)\left(\sigma_{I}^{2}+\sigma_{I_{ref}}^{2}+c_{2}\right)}$$

where subscripts *I* and *I_ref_* represent the reconstructed image and the reference image, respectively, and *μ* and *σ* are the mean and standard variance of the image, respectively. *c*_1_ = (0.01 × *R*)^2^, *c*_2_ = (0.03 × *R*)^2^ are two constant terms to prevent the denominator from equaling 0, and *R* is the range of pixel values of the image. The SSIM is closer to 1, meaning that the reconstructed image is of better quality. The RMSE directly reflects the pixel distance error between the reconstructed image and the reference image

(21) $$\mathrm{RMSE}=\sqrt{\frac{1}{N^{2}}{\left\|I-I_{ref}\right\|}_{2}^{2}}$$

where *N* is the width or height of the image, and a smaller RMSE value means that the reconstructed image is closer to the reference image. PSNR measures the proportion of useful information in an image by calculating the ratio of the energy of the peak signal to the average energy of the noise

(22) $$\mathrm{PSNR}=20\times \log_{10}\left(\frac{\mathrm{MAX}\left(I,I_{ref}\right)}{\mathrm{RMSE}}\right)$$

where MAX(*I*, *I_ref_*) is the maximum value between the image and the reference image. A higher PSNR means that the image has less noise and is of higher quality.

The means and standard deviation distributions of PSNR, SSIM, and RMSE for the reconstruction results obtained using various methods with different sparse projection views are listed in Table 1. The quantitative evaluation results indicate that the results of the FBP are the worst for all sparse view conditions, and the results of the post-processing methods (MWNet) are lower than those of other hybrid domain and iteration reconstruction networks in all sparse view cases. Further, DDPTransformer can capture richer edge feature information of CT images by combining different patch segmentation schemes on top of SwinT, which makes it further superior to DuDoTrans in 128 and 32 views cases. In terms of both 64 and 32 views, MIST achieves the second-best quantitative evaluation results among all methods due to the use of multiple UNets to iteratively recover sinograms and CT images in different domains and the combination of Transformer to further refine the quality of the CT images, resulting in better reconstruction performance. In addition, due to the limitations of the memory resources of the GPU, we only set up nine iterative layers in RegFormer, which may have made its performance degrade, but it still maintains a relatively stable result. Our method further combines recurrent convolution and wavelet transform and enhances the reconstruction performance of sinograms and CT images by fusing and recovering distinct frequency features through separate network branches and self-attention. In comparison to MIST, our method achieves the best quantitative evaluation results while avoiding the iterative use of network modules that lead to increased parameters. Specifically, in the cases of 128, 64, and 32 projection views, our method gains improvements of 2.9408 dB, 1.7121 dB, and 1.0393 dB for PSNR to MIST, respectively.

**Figure 8:**
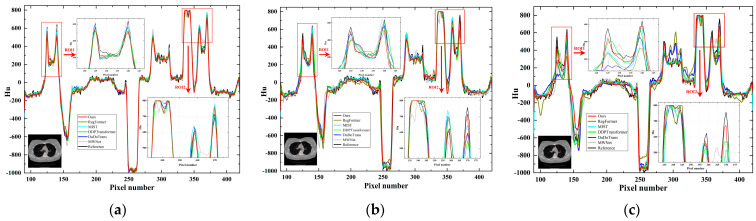
Intensity profile results for different methods corresponding to the red vertical line with different projection views. (**a**) 128 projection views; (**b**) 64 projection views; (**c**) 32 projection views.

Further, the line intensity profiles in the CT images reconstructed using different methods with different sparse projection view cases are displayed in Figure 8. From the observations in the enlarged ROI1 and ROI2 in Figure 8, it becomes evident that as the number of projection views decreases, the line intensity profiles of the other comparison methods produce larger deviations from the reference profiles. Our method, however, is still able to maintain results closer to the reference profiles, even though the number of sparse views is only 32. In the overall case, our method demonstrates a more stable and superior reconstruction performance for edge information. When the number of projection views is less, our method exhibits superior performance compared to other methods.

### 3.4. Robustness of Noise

**Figure 9:**
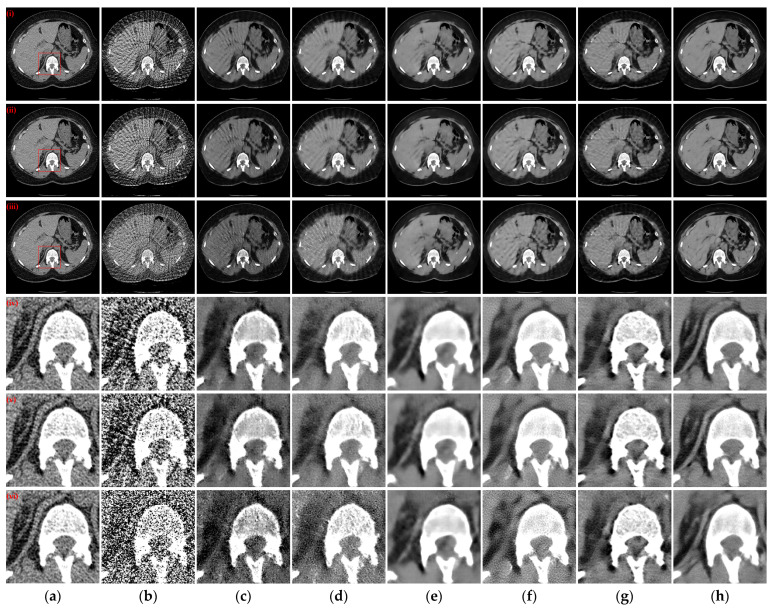
The SVCT reconstruction results from different methods for different intensity levels of mixed noise with 64 projection views. (**a**) The reference image; (**b**) FBP; (**c**) MWNet; (**d**) DuDoTrans; (**e**) DDPTransformer; (**f**) MIST; (**g**) RegFormer; (**h**) Ours. (**i**)–(**iii**) are the reconstructed CT images with Poisson noise levels of 1 × 10^6^, 5 × 10^5^, and 1 × 10^5^, respectively, and (**iv**)–(**vi**) correspond to the enlarged ROIs marked by the red boxes in the reference images above. The display window is [−160, 240] HU.

In practical CT reconstruction systems, the acquisition of X-ray projection data by the detector is subject to photon noise, resulting in noise in the reconstructed CT images and the subsequent degradation of the quality of SVCT images. The robustness of the model to noise is crucial for practical applications. Therefore, in the case of 64 projection views, different intensity levels of “Gaussian + Poisson” noise are added to the sinograms according to Equations (16)–(18) to simulate the generation process of photon noise. Specifically, Poisson noise with average photon counts *I*_0_ = 1 × 10^6^, 5 × 10^5^, and 1 × 10^5^ and Gaussian noise with mean *m* = 0 and variance *var* = 0.05 are added to the sinograms, respectively. Subsequently, without retraining the model, the previously trained models are directly used to reconstruct the sinograms with varying intensity levels of mixed noise. Figure 9 illustrates the reconstruction results from various methods for different intensity levels of mixed noise with 64 projection views, including reconstructed CT images and enlarged ROIs.

**Figure 10:**
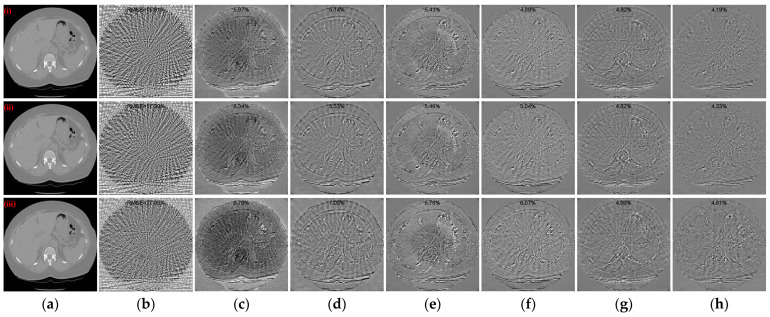
Absolute difference images relative to the reference image with different noise levels. (**a**) The reference image; (**b**) FBP; (**c**) MWNet; (**d**) DuDoTrans; (**e**) DDPTransformer; (**f**) MIST; (**g**) RegFormer; (**h**) Ours. (**i**)–(**iii**) correspond to the results with the Poisson noise levels of 1 × 10^6^, 5 × 10^5^, and 1 × 10^5^, respectively. The display window is [−180, 180] HU.

The observations from Figure 9(i)–(iii) indicate that as the intensity level of mixed noise added to the sinograms increases, more noise is generated in the reconstructed CT image. Especially when the Poisson noise level is 1 × 10^5^, the area of structural blur increases, while both DDPTransformer and our method recover clearer structures. The enlarged ROI results in Figure 9(iv)–(vi) show that DuDoTrans produces many tiny spurious structures with Photon noise level of 1 × 10^5^. MIST recovers better results for Poisson noise levels of 1 × 10^6^ and 5 × 10^5^, but its recovery performance decreases rapidly when the Poisson noise level increases to 1 × 10^5^. In contrast, with our method, DDPTransformer and RegFormer still recover better results. Moreover, our method maintains the best qualitative results in all cases. Figure 10 shows the absolute difference images and rRMSE value of the reconstruction results obtained using different methods. Further insights from the results in Figure 10(iii) highlight that the reconstructed images obtained based on our method lose less structural information and have the best rRMSE values, even in the case of a Poisson noise level of 1 × 10^5^.

Further, the quantitative evaluation metrics for the reconstruction results are listed in Table 2, including the means and standard deviation distributions of PSNR, SSIM, and RMSE for the reconstruction results of all methods with different noise intensity levels of mixed noise. Similarly, the reconstruction performance of MIST degrades rapidly when a mixed noise with an average photon count of 1 × 10^5^ is added. Although DDPTransformer and RegFormer exhibit lower reconstruction performance with Poisson noise levels of 1 × 10^6^ and 5× 10^5^, they maintain more stable reconstruction results compared to other DL-based methods, resulting in better quantitative evaluation metrics than MIST when reconstructing at a Poisson noise level of 1 × 10^5^. Notably, our method consistently achieves the best quantitative results in all noise experiments with different intensity levels.

### 3.5. Ablation Study

In order to verify the effectiveness of the different modules in the proposed dual-domain reconstruction network and edge loss, different ablation experiments were conducted with 64 sparse projection views. Eight different dual-domain networks were trained by removing or replacing different modules to assess the impact of different improved structures on the reconstruction quality of the CT image. The first model serves as the baseline, where the Conv-LSTM is substituted with the standard feature extraction module (CBR) and the adaptive soft thresholding function is replaced with the ReLU activation function while maintaining the presence of the SNFM. Model 2 then verifies the effectiveness of the ACSTF by replacing the ReLU activation function with it in the baseline. Model 3 replaces the CBR in the baseline with the Conv-LSTM to verify the impact of Conv-LSTM on model performance. The fourth model uses a 3 × 3Conv-based Conv-LSTM based on Model 3 and replaces the ReLU activation function with the ACSTF. To further confirm the enhanced effectiveness of the improved MFNF block, a comparison was conducted involving the self-attention calculation in the SwinT, and Model 5 was trained in this context. For Models 6 and 7, the fusion strategy in SNFM was changed to summation fusion and concatenation fusion, respectively. Finally, to verify the impact of different wavelet transforms on the reconstruction performance of the proposed model, Model 8 replaces the Harr wavelet transform with the Daubechies wavelet transform [48], and the filter length of the Daubechies wavelet transform is set to four.

**Figure 11:**
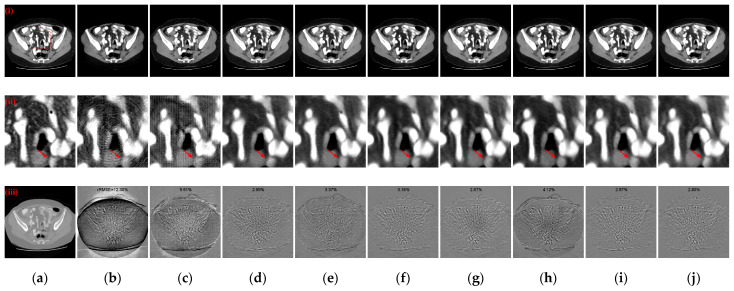
Qualitative comparative results of different ablation models. (**a**) The reference image; (**b**) baseline; (**c**) Model 2; (**d**) Model 3; (**e**) Model 4; (**f**) Model 5; (**g**) Model 6; (**h**) Model 7; (**i**) Model 8; (**j**) Ours. (**i**)–(**iii**) are reconstructed CT images, enlarged ROIs, and absolute difference images, respectively. The display window for the reconstructed CT images and enlarged ROIs is [−160, 240] HU; the display window for absolute difference images is [−180, 180] HU.

The quantitative evaluation metrics of the reconstruction results of the eight ablation models and the proposed complete dual-domain network are presented in Table 3. The results in Table 3 indicate that the incorporation of the Conv-LSTM and ACSTF in the baseline (Model 2 and Model 3) leads to enhancements in the reconstruction performance. In particular, the ACSTF exhibits a more significant improvement in the PSNR values of the reconstruction results, underscoring its ability to enhance the denoising performance of the model. This improvement is achieved with a relatively minor increase in the number of parameters, totaling 0.45 M. Furthermore, compared to Model 4, an enhancement is made to the Conv-LSTM by employing Dconv to decrease the number of parameters. This modification not only effectively reduces the number of parameters but also contributes to further improvements in CT reconstruction performance. Additionally, the DConv-based SNFM fusion strategy yields superior reconstruction metrics compared to the self-attention fusion strategies based on SwinT, as observed in the results of Model 5. Moreover, our feature fusion approach demonstrates superior results compared to simple summation or concatenation fusion strategies (Model 6 and Model 7), all while maintaining a lower parameter count than channel concatenation fusion strategy. Finally, compared to Daubechies wavelet transform (Model 8), the Haar wavelet transform-based dual-domain network achieves higher quantitative evaluation metrics. It illustrates that although the Daubechies wavelet has superior smoothness and adaptability compared to Haar wavelet, it lacks symmetry and produces phase distortion when reconstructing the signal, which may increase deviation between the reconstruction result and the reference image. Figure 11 shows the qualitative comparative results of the above different ablation experimental models, including reconstructed CT images, enlarged ROIs, and absolute difference images. As observed in the enlarged ROIs and absolute difference images in Figure 11(ii,iii), the complete dual-domain model has higher structural fidelity, with less structural information lost in the absolute difference images.

Additionally, we conducted a separate analysis by independently training SDNet and IDNet to assess the SVCT reconstruction performance of the distinct domain sub-networks. The reconstruction results of two sub-networks and the complete dual-domain network are quantitatively evaluated and presented in Table 4. Notably, the evaluation metrics for IDNet surpass those of SDNet. This observation indicates that our proposed network structure based on wavelet transform and recurrent convolution has more powerful performance in capturing redundant and repeated feature information in sinograms. However, the performance of both is inferior to that of the dual-domain network.

**Figure 12:**
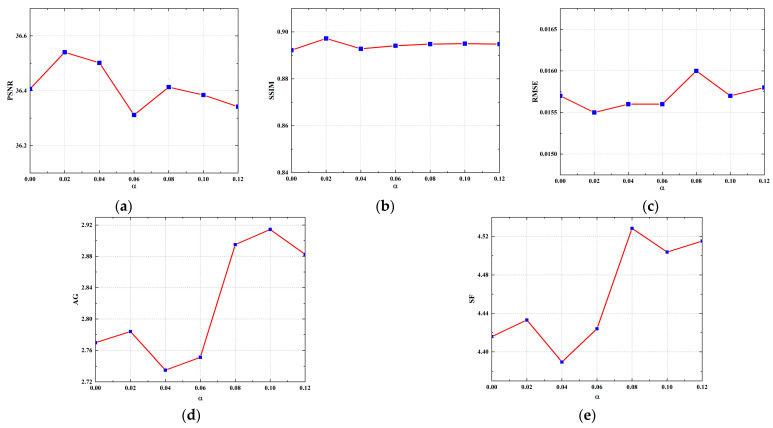
Ablation experiments for edge loss with different weight parameters. (**a**) Line graph of PSNR value; (**b**) line graph of SSIM value; (**c**) line graph of RMSE value; (**d**) line graph of AG value; (**e**) line graph of SF value.

To further assess the effectiveness of the designed edge loss and determine the optimal weight parameter for the loss, the model was retrained by varying the weight parameter for the edge loss. Subsequently, these retrained models were quantitatively evaluated using the test set. Additionally, to quantitatively evaluate the model’s ability to reconstruct edge information, in addition to using the PSNR, SSIM, and RMSE metrics, we incorporated the average gradient (AG) and spatial frequency (SF) metrics [49] to evaluate the edge quality of the CT images. The larger the AG and SF values, the more detailed the information at the edges of the image, and the sharper it is. The line graphs in Figure 12 depict the PSNR, SSIM, RMSE, AG, and SF values for the reconstruction results of the models trained with different weight parameters using 64 projection views. As can be observed from Figure 12, the inclusion of the edge loss regularization term to the MSE loss consistently enhanced the reconstruction performance of the model. A larger weight parameter (α) for the edge loss leads to higher mean values of AG and SF, indicating the improved ability of the model to reconstruct edge and detail information of the image. However, excessively large α results in a decrease in the PSNR value and an increase in the RMSE value. It is possible that over-enhancing the model’s ability to reconstruct the edge structure causes its reconstructed edge pixels to have a large deviation from the label. Notably, when α is set to 0.02, improvements are observed in all metrics. This indicates that the optimal weight parameter for the edge loss was determined to be 0.02.

### 3.6. Computational Cost

To assess and compare the computational resource requirements of the different networks, details regarding the total number of parameters, floating point operations (FLOPs), and running time for all DL-based reconstruction methods are provided in Table 5, using 128 projection views. Specifically, the initial number of channels of the image domain network in the DDPTransformer is expanded to 64 to enhance the performance of the model, and parameter-independent UNets are employed for the MIST. Due to the limitations of the memory resources of the GPU, we only set the depth and number of Transformer heads to 2 for the SwinT block in the MIST model. Similarly, the number of iterative layers is only set to 9 for the RegFormer model.

As indicated in Table 5, MWNet (post-processing methods) has a relatively large number of parameters due to the stacking of excessively deep convolutional layers. In the hybrid domain model, MIST achieves superior reconstruction results compared to DuDoTrans and DDPTransformer. However, the multiple iterative use of UNet results in an excessive number of parameters for the MIST model. In contrast, our method not only enhances the reconstruction performance but also reduces the number of model parameters compared to MIST and MWNet, albeit with an increased computational overhead. Furthermore, we compare the average running time of different models for processing a single slice of the test set on an NVIDIA RTX 3090 GPU, with the batch size set to 1. As presented in the FLOPs and running time results in Table 5, despite our proposed model using DConv as the base feature extraction block to reduce the number of model parameters, the grouped convolution operation of DConv in PYTorch faces challenges related to low computational parallelism. The excessive use of DConv also increase the memory access cost of the model [50], resulting in a slower runtime and higher FLOPs for our model compared to the post-processing network and other hybrid domain networks. However, when compared to MIST, our model greatly improves the reconstruction performance of the model with a minor increase in running time. Moreover, as demonstrated in the experimental results in Table 2 and Figure 9, the reconstruction performance of MIST decreases significantly as the noise level increases, while our model maintains the best qualitative and quantitative evaluation results, proving more effective at suppressing noise. Finally, our method also exhibits a lower running time compared to the RegFormer iteration model and shows excellent performance.

## 4. Discussion

**Figure 13:**
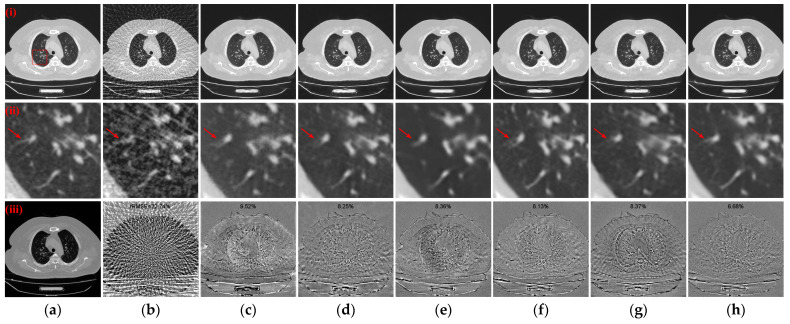
Qualitative comparative results of different models on the COVID-19-CT dataset with 64 projection views. (**a**) The reference image; (**b**) FBP; (**c**) MWNet; (**d**) DuDoTrans; (**e**) DDPTransformer; (**f**) MIST; (**g**) RegFormer; (**h**) Ours. (**i**)–(**iii**) are reconstructed CT images, enlarged ROIs, and absolute difference images, respectively. The display window for the reconstructed CT images and enlarged ROIs is [−1200, 300] HU; the display window for absolute difference images is [−180, 180] HU.

In Section 3, the experimental results based on the “LDCT and-Projection-data” dataset affirm that our approach outperforms other DL-based reconstruction algorithms in terms of both qualitative and quantitative evaluation across various sparse projection views. Numerical simulation experiments with different noise intensity levels further confirm the robustness of our algorithm to photon noise. Additionally, the effectiveness of the RCU, SNFM, ACSTF, and edge loss function is demonstrated by the results of a series of ablation models. Further, the generalizability of the different trained models is tested using slices from another patient in the COVID-19-CT dataset [51] with 64 projection views. These CT slices were provided by two main general university hospitals in Mashhad and Iran, where the data were scanned under the protocol of 120 kVp and 80 effective mAs. The test set contains 425 lung CT images, with each CT being composed of 512 × 512 pixels. Table 6 lists the means and standard deviation distributions of quantitative metrics for the different models on the COVID-19-CT dataset with 64 projection views. As observed in Table 6, there is a decrease in quantitative metrics for all models without retraining. Among the compared methods, the MIST the RegFormer demonstrates better generalizability to different datasets. Notably, our model maintains the best quantitative metrics compared to other DL-based models. Specifically, compared to the second-best result, our model improves the PSNR and SSIM metrics by 1.5098 dB and 0.0235, respectively. Figure 13 illustrates the qualitative comparative results of the different models, including reconstructed CT images, enlarged ROIs, and absolute difference images. As shown in the ROIs in Figure 13(ii), our model performs well in handling details such as lung texture, streak artifacts, and preserving more structures. Additionally, as shown in the absolute difference images in Figure 13(iii), our model achieves the best results in both qualitative and rRMSE. In line with the quantitative comparison, our model shows better reconstruction in both global structure and detailed information.

Although our method achieves a significant improvement in reconstruction performance, the extensive use of DConv leads to a decrease in the computational parallelism of the model, thereby increasing the time required for CT reconstruction. Therefore, building upon the insights gained from this study, our focus will extend to the development of a streamlined Transformer-based feature extraction module. This innovative design aims to bolster the model’s effectiveness in capturing comprehensive global feature information from high-resolution feature maps while maintaining a lightweight architecture to increase the computational speed of the model. Additionally, the simple Haar wavelet transform may exhibit limited smoothness in resolution transition for signals. As shown in Figure 6, the reconstruction results suffer from significant structural blurring when the projection views are extremely sparse. Therefore, we will also study the effect of more different wavelet transforms on the decomposition of sinograms and CT images to improve the reconstruction performance of the model for different frequency components.

## 5. Conclusions

In this paper, a dual-domain reconstruction network incorporating multi-level wavelet transform and recurrent convolution is proposed for SVCT imaging. Two-dimensional DWT is employed to decompose sinograms and CT images into distinct frequency bands. The texture information in distinct frequency components is then recovered through different network branches. Our novel Conv-LSTM-based RCU effectively captures overarching features from sinograms and global artifacts present in CT images. It adeptly models long-range dependencies existing between different feature maps. In the high-frequency component recovery branches of the model, the alignment and fusion of high- and low-frequency features are achieved through the SNFM, effectively enhancing the recovery of high-frequency components. Moreover, the application of ACSTF removes redundant noise features. Finally, the reduction in structural and texture blurring in CT images is accomplished through the incorporation of edge loss as a regularization term within the image domain loss function. The experimental results demonstrate that our proposed model is suitable for different sparse views and human organs and effectively removes more artifacts and preserves more structural information. The proposed method also achieves the best results in different quantitative metrics compared with other DL-based reconstruction algorithms. Further, the generalizability of our model to different datasets is demonstrated by testing it on the COVID-19-CT dataset. Moving forward, our future endeavors will further explore extensions of the network to enhance both the reconstruction speed and performance of the model, especially under extremely sparse views.

## Figures and Tables

**Table 1:** Quantitative evaluation results of different methods. The best results are marked in bold, and the second-best results are underlined.

Method	128 Projection Views			64 Projection Views			32 Projection Views		
	PSNR	SSIM	RMSE	PSNR	SSIM	RMSE	PSNR	SSIM	RMSE
FBP	17.0616 ± 1.6025	0.4801 ± 0.0486	0.1424 ± 0.0223	14.0979 ± 1.2326	0.3835 ± 0.0480	0.1991 ± 0.0253	11.8461 ± 1.1073	0.3189 ± 0.0473	0.2577 ± 0.0031
MWNet	35.3025 ± 2.0618	0.8671 ± 0.0542	0.0177 ± 0.0041	32.3812 ± 1.7980	0.8195 ± 0.0480	0.0246 ± 0.0060	28.6675 ± 2.1581	0.7816 ± 0.0563	0.0379 ± 0.0085
DuDoTrans	37.5434 ± 2.9086	0.8968 ± 0.0407	0.0138 ± 0.0034	33.5129 ± 2.1423	0.8489 ± 0.0443	0.0216 ± 0.0042	29.3935 ± 1.9850	0.7978 ± 0.0493	0.0347 ± 0.0071
DDPTrans	37.9856 ± 1.2154	0.9034 ± 0.0081	0.0138 ± 0.0037	33.3891 ± 2.1731	0.8443 ± 0.0511	0.0216 ± 0.0047	30.6977 ± 1.8773	0.8175 ± 0.0607	0.0298 ± 0.0056
MIST	36.7952 ± 1.9551	0.9059 ± 0.0374	0.0148 ± 0.0029	34. 6944 ± 2.0515	0.8768 ± 0.0482	0.0189 ± 0.0040	30.9089 ± 1.5734	0.8286 ± 0.0489	0.0289 ± 0.0050
RegFormer	37.3118 ± 2.1226	0.8897 ± 0.0418	0.0141 ± 0.0031	33.5659 ± 1.6660	0.8471 ± 0.0463	0.0214 ± 0.0038	30.7162 ± 1.6209	0.8059 ± 0.0535	0.0296 ± 0.0050
Ours	**39.7360 ± 3.1812**	**0.9279 ± 0.0356**	**0.0108 ± 0.0034**	**36.4065 ± 2.5655**	**0.8922 ± 0.0468**	**0.0157 ± 0.0040**	**31.9482 ± 1.8559**	**0.8438 ± 0.0514**	**0.0258 ± 0.0052**

**Table 2:** Quantitative evaluation results of different methods with different intensity noise levels. The best results are marked in bold, and the second-best results are underlined.

Method	Noise-L1 (1 × 10^6^)			Noise-L2 (5 × 10^5^)			Noise-L3 (1 × 10^5^)		
	PSNR	SSIM	RMSE	PSNR	SSIM	RMSE	PSNR	SSIM	RMSE
FBP	14.0077 ± 1.1170	0.3427 ± 0.0348	0.2009 ± 0.0239	13.9236 ± 1.0353	0.3082 ± 0.0281	0.2027 ± 0.0229	13.2971 ± 0.7244	0.2017 ± 0.0228	0.2171 ± 0.0180
MWNet	32.1469 ± 1.3300	0.8141 ± 0.0491	0.0250 ± 0.0038	31.6998 ± 1.1159	0.7972 ± 0.0469	0.0262 ± 0.0033	28.4697 ± 0.8459	0.6143 ± 0.0371	0.0379 ± 0.0040
DuDoTrans	33.3070 ± 2.9086	0.8410 ± 0.0428	0.0221 ± 0.0042	33.0065 ± 1.8279	0.8275 ± 0.0401	0.0228 ± 0.0041	30.6072 ± 1.2689	0.6887 ± 0.0377	0.0298 ± 0.0036
DDPTrans	33.3621 ± 2.1675	0.8441 ± 0.0596	0.0220 ± 0.0047	33.3207 ± 2.1778	0.8437 ± 0.0584	0.0221 ± 0.0047	32.8704 ± 1.8773	0.8405 ± 0.0590	0.0232 ± 0.0056
MIST	34.4409 ± 1.9623	0.8699 ± 0.0473	0.0194 ± 0.0039	34. 0791 ± 2.0515	0.8575 ± 0.0448	0.0202 ± 0.0038	32.1646 ± 1.3665	0.7811 ± 0.0366	0.0250 ± 0.0035
RegFormer	33.4319 ± 1.8588	0.8465 ± 0.0463	0.0218 ± 0.0044	33.3905 ± 1.8442	0.8451 ± 0.0463	0.0219 ± 0.0044	33.0737 ± 1.6209	0.8339 ± 0.0535	0.0227 ± 0.0050
Ours	**36.2235 ± 2.4382**	**0.8915 ± 0.0356**	**0.0159 ± 0.0039**	**35.7969 ± 2.5655**	**0.8822 ± 0.0436**	**0.0167 ± 0.0038**	**34.3397 ± 2.0856**	**0.8436 ± 0.0359**	**0.0197 ± 0.0041**

**Table 3:** Quantitative evaluation results for different ablation models. The best results are marked in bold, and the second-best results are underlined.

		Baseline	Model 2	Model 3	Model 4	Model 5	Model 6	Model 7	Model 8	Ours
Network Structure	Conv-LSTM	CBR	CBR	√	3 × 3Conv-based	√	√	√	√	√
	ACSTF	×	√	×	√	√	√	√	√	√
	SNFM	√	√	√	√	SwinT	×	×	√	√
	Summation fusion	×	×	×	×	×	√	×	×	×
	Concatenation fusion	×	×	×	×	×	×	√	×	×
	Haar wavelet transform	√	√	√	√	√	√	√	Daubechies wavelet transform	√
PSNR		29.8588 ± 1.2407	31.0563 ± 1.5195	35.9344 ± 2.1782	36.2666 ± 2.3966	35.4864 ± 2.1166	36.1024 ± 2.2599	33.2770 ± 1.9848	36.2853 ± 2.5442	**36.4065 ± 2.5655**
SSIM		0.7239 ± 0.0437	0.7429 ± 0.0307	0.8733 ± 0.0467	0.8883 ± 0.0467	0.8727 ± 0.0495	0.8864 ± 0.0430	0.8586 ± 0.262	0.8918 ± 0.0472	**0.8922 ± 0.0468**
RMSE		0.0325 ± 0.0045	0.0285 ± 0.0057	0.0164 ± 0.0037	0.0159 ± 0.0038	0.0173 ± 0.0038	0.0162 ± 0.0038	0.0222 ± 0.0024	0.0159 ± 0.0047	**0.0157 ± 0.0040**
Param (M)		16.68	17.13	20.03	54.01	20.90	**14.26**	22.29	20.47	20.47

**Table 4:** Quantitative evaluation results for different sub-networks. The best results are marked in bold, and the second-best results are underlined.

Method	PSNR	SSIM	RMSE
SDNet	34.3578 ± 2.6077	0.8661 ± 0.0499	0.0199 ± 0.0049
IDNet	32.9327 ± 1.7221	0.8496 ± 0.0512	0.0230 ± 0.0044
Ours	**36.4065 ± 2.5655**	**0.8922 ± 0.0468**	**0.0157 ± 0.0040**

**Table 5:** Computational costs of different DL-based reconstruction methods. The best results are marked in bold, and the second-best results are underlined.

	MWNet	DuDoTrans	DDPTrans	MIST	RegFormer	Ours
Param (M)	31.21	**0.62**	0.95	27.30	2.41	20.47
FLOPs (G)	**87.52**	125.51	182.24	615.13	631.70	751.59
Time (ms)	**17.53**	159.86	60.95	262.82	334.70	284.77

**Table 6:** Quantitative evaluation results for different models on the COVID-19-CT dataset with 64 projection views. The best results are marked in bold, and the second-best results are underlined.

	FBP	MWNet	DuDoTrans	DDPTrans	MIST	RegFormer	Ours
PSNR	20.0494 ± 1.0089	28.8013 ± 1.5426	30.2586 ± 1.4001	29.8582 ± 1.3986	30.3650 ± 1.3708	30.4801 ± 1.9848	**31.9899 ± 1.8273**
SSIM	0.3179 ± 0.0312	0.7356 ± 0.0747	0.7608 ± 0.0671	0.7325 ± 0.0916	0.7683 ± 0.0729	0.7584 ± 0.0764	**0.7918 ± 0.724**
RMSE	0.1001 ± 0.0012	0.0369 ± 0.0090	0.0311 ± 0.0031	0.0325 ± 0.0057	0.0307 ± 0.0053	0.0303 ± 0.0031	**0.0257 ± 0.0072**

## Data Availability

The “Low Dose CT Image and Projection Data (LDCT-and-Projection-data)” dataset can be downloaded at https://doi.org/10.7937/9npb-2637 (accessed on 3 April 2023). The COVID-19-CT dataset can be downloaded at https://doi.org/10.7910/DVN/6ACUZJ (accessed on 30 December 2023).
